# Metabolic Engineering *Kluyveromyces marxianus* for Isoprene Production from *Eucalyptus globulus* Wood Cellulosic Fraction

**DOI:** 10.3390/jof12050343

**Published:** 2026-05-06

**Authors:** Marlene Baptista, Jean-Marc Daran, Lucília Domingues

**Affiliations:** 1 CEB—Centre of Biological Engineering, University of Minho, Campus Gualtar, 4710-057 Braga, Portugal; malenebq16@gmail.com; 2 Department of Biotechnology, Delft University of Technology, 2628 Delft, The Netherlands; j.g.daran@tudelft.nl; 3 LABBELS—Associate Laboratory, 4710-057 Braga, Portugal

**Keywords:** isoprene, isoprene synthase, *Kluyveromyces marxianus*, mevalonate pathway, *Eucalyptus globulus* wood

## Abstract

Isoprene, a highly volatile hydrocarbon with numerous industrial applications, has traditionally been produced from petrochemical sources through processes associated with significant environmental impacts. Microbial production of isoprene has emerged as a promising and more sustainable alternative. In this study, the potential of the non-conventional yeast *Kluyveromyces marxianus* to produce isoprene from a renewable feedstock was explored, contributing to the development of a more sustainable process. *K. marxianus* was engineered to produce isoprene from glucose through the expression of an isoprene synthase gene, and the gene copy number of this enzyme was found to significantly influence isoprene production. Furthermore, enhancing the supply of the isoprene precursors acetyl-CoA and dimethylallyl diphosphate (DMAPP) via engineering of the mevalonate pathway led to increased production. A higher headspace-to-culture ratio in sealed serum bottles also facilitated isoprene accumulation. Importantly, isoprene production was achieved from the cellulosic fraction of pretreated *Eucalyptus globulus* wood. To our knowledge, this is the first report of isoprene production in *K. marxianus* using a lignocellulosic feedstock, providing proof of concept for its potential in integrated processes based on sustainable substrates under stressful conditions.

## 1. Introduction

The growing global population has driven an increasing demand for rubber across several industries, including automotive, construction, aerospace, and healthcare. In 2020, global rubber production was approximately 24 million tons, with synthetic rubber accounting for about 60% of this total [1]. Synthetic rubber encompasses a range of materials, including styrene-butadiene rubber, butyl rubber, and polybutadiene, among others. Polyisoprene—a synthetic analogue of natural rubber—is produced from isoprene (C_5_H_8_) monomers, a highly volatile hydrocarbon identified as one of the top chemical targets for production from biorefinery-derived carbohydrates [2]. Although global isoprene production is around one million tons per year, with 95% used for polyisoprene manufacture, polyisoprene represents only a small portion of the total synthetic rubber market. Isoprene encounters other applications as an intermediate in the pharmaceutical, cosmetic, and chemical industries [3,4,5]. The isoprene market has been growing strongly, and it is expected to reach USD 4.64 billion by 2028, representing a compound annual growth rate (CAGR) of 8.2% [6]. Currently, isoprene is produced almost entirely from petrochemical sources, which are intensive and environmentally unfriendly processes that release intense greenhouse gas emissions (0.65 kg CO_2_ kg^−1^ of isoprene) [7,8]. Moreover, these processes are influenced by fluctuations in the petroleum price, require high energy input, involve complex purification steps, and result in low yields [9].

Microbial production of isoprene by bacteria and yeast has emerged as a sustainable, viable alternative to traditional chemical synthesis routes [10,11]. This has been achieved by the introduction of heterologous isoprene synthases (IspSs) from plants, which directly convert dimethylallyl diphosphate (DMAPP) into isoprene [12,13]. These enzymes compete with DMAPP and isopentenyl diphosphate (IPP) utilization for the generation of geranyl diphosphate (GPP) and farnesyl diphosphate (FPP), which are key for synthesizing molecules involved in cellular maintenance, such as sterols [14].

Considering the current need to shift to sustainable manufacturing processes, lignocellulosic materials have been largely exploited as alternative feedstocks for fuels and chemicals production due to their abundance and global availability. These materials are generally composed of 30–60% (on a dry basis) cellulose, 20–40% hemicellulose, and 10–25% lignin [15]. *Eucalyptus globulus* wood is the most widely available lignocellulosic feedstock in Portuguese paper mills, and it has a high percentage of cellulose compared to other materials [16]. Breaking down the recalcitrant structure of lignocellulosic biomass through a pretreatment allows the recovery of each component either in solid (cellulosic fraction) or liquid (hemicellulosic fraction) phases, thereby enhancing the accessibility of carbohydrates for subsequent enzymatic or acid hydrolysis [17].

The non-conventional yeast *Kluyveromyces marxianus*, commonly found in fermented dairy products [18], has gained recognition as a promising alternative cell factory to produce ethanol, high-value chemicals, and heterologous enzymes. Its versatile applications span various industries, including food, feed, and pharmaceutical [19]. In addition to its rapid growth rate among eukaryotes and thermotolerance, often advantageous for processes combining saccharification and fermentation, *K. marxianus* can metabolize a variety of carbon sources found in lignocellulosic feedstocks, including glucose, xylose, and arabinose. As such, this yeast is recognized as a promising microbial cell factory for the valorization of lignocellulosic biomass [20,21]. Recently, the first work on terpenoid production in *K. marxianus* was published, showing the ability of this yeast to produce sabinene [22]. Carotenoid production by *K. marxianus*, which requires mevalonate (MVA) pathway precursors from a sustainable feedstock, has also been reported [23]. While the potential of *K. marxianus* for isoprenoid production remains largely unexplored, existing reports suggest that this yeast could serve as a promising chassis for isoprene biosynthesis via the MVA pathway.

Given the potential of *K. marxianus* to produce acetyl-CoA-derived compounds, isoprene production by this yeast was assessed for the first time. Additional metabolic engineering strategies targeting the MVA pathway, along with optimization of culture conditions, were explored to improve isoprene production. Finally, sustainable isoprene production was achieved using cellulosic hydrolysates derived from *E. globulus* wood.

## 2. Materials and Methods

### 2.1. Media and Cultivation

*Escherichia coli* transformants were selected and maintained on lysogeny broth plates (1% NaCl (Labkem, Dublin, Ireland), 1% peptone (Liofilchem, Roseto degli Abruzzi, Italy), 0.5% yeast extract (Liofilchem, Roseto degli Abruzzi, Italy), and 1.5% agar (Liofilchem, Roseto degli Abruzzi, Italy)) with the appropriate antibiotic (100 mg L^−1^ of ampicillin, 50 mg L^−1^ of kanamycin or 50 mg L^−1^ of chloramphenicol). YPD medium plates (1% yeast extract (Liofilchem, Roseto degli Abruzzi, Italy), 2% peptone (Liofilchem, Roseto degli Abruzzi, Italy), 2% dextrose (Sigma-Aldrich, St. Louis, MO, USA), 1.5% agar (Liofilchem, Roseto degli Abruzzi, Italy) with the appropriate selection (200 mg L^−1^ of hygromycin B (hygB; Roche, Switzerland) and G418 (Fisher Scientific, Waltham, MA, USA) or 50 mg L^−1^ of nourseothricin (nat; Jena Bioscience, Germany) were used for yeast selection and maintenance. For selection of strains harbouring plasmid pKlNatCre, YPG medium (1% yeast extract (Liofilchem, Roseto degli Abruzzi, Italy), 2% peptone (Liofilchem, Roseto degli Abruzzi, Italy), 2% galactose (Sigma-Aldrich, St. Louis, MO, USA)) was used.

Selection of uracil auxotrophic strains was performed in synthetic defined (SD) medium plates containing 6.7% yeast nitrogen base without amino acids (YNB; Sigma-Aldrich, St. Louis, MO, USA), 5% glucose (NZYTech, Lisbon, Portugal), 1.5% agar (Liofilchem, Roseto degli Abruzzi, Italy), and 1x amino acid mix supplemented with 1 g L^−1^ of 5-fluoroorotic acid (FOA; UPS Biologics, Louisville, KY, USA). The amino acid mix was composed of 1.7 g L^−1^ of L-arginine (Sigma-Aldrich, St. Louis, MO, USA), 2.7 g L^−1^ of L-aspartic acid (Merck, Darmstadt, Germany), 1.7 g L^−1^ of L-isoleucine (Acros Organics, Geel, Belgium), 1.7 g L^−1^ of DL-Lysine monohydrate (Alfa Aesar, Lancashire, UK), 0.7 g L^−1^ of L-methionine (Acros Organics, Geel, Belgium), 1.7 g L^−1^ of L-phenylalanine (Alfa Aesar, Lancashire, UK), 3.3 g L^−1^ of L-threonine (Sigma-Aldrich, St. Louis, MO, USA), 1.7 g L^−1^ of L-tyrosine (Honeywell Fluka, Seelze, Germany), 4.7 g L^−1^ of L-valine (Merck, Darmstadt, Germany), 0.7 g L^−1^ of L-histidine (Fisher Scientific, Waltham, MA, USA), 3.3 g L^−1^ of L-leucine (Fisher Scientific, Waltham, MA, USA), and 1.7 g L^−1^ of L-tryptophan (Merck, Darmstadt, Germany). Also, SD medium contains 0.3 g L^−1^ of adenine (Alfa Aesar, Lancashire, UK).

### 2.2. Construction of Plasmids and Strains

All *K. marxianus* strains (Supplementary Table S1) and plasmids (Supplementary Table S2) constructed in this work, as well as the oligonucleotides (Supplementary Table S3) used, are listed in Supplementary Materials. Cloning procedures were done using *E. coli* NZY5α competent cells (NZYTech, Lisbon, Portugal). Yeast transformation was done following the lithium acetate/PEG method using 2 µg of integrative cassette after digestion with *Not*I (Thermo Scientific, Waltham, MA, USA) [24]. When using the CRISPR-Cas9 system, 500 ng of plasmid and 1 µg of cassette were transformed. Plasmids construction was made by Golden Gate using YTK parts (Addgene, Waltham, MA, USA) [25,26] and some parts from the *Kluyveromyces* kit [27]. Some plasmids were provided by others, including pGGKd015 [28], pGGKd072 [29], pGGkd068 [30], and pSphI [31].

Chloroplast transition peptide was removed from the chemically synthesized *Populus alba* isoprene synthase (IspS) sequence, codon-optimized for expression in *K. marxianus*, according to prediction in TargetP online server, version 2.0.

A level I plasmid part harbouring the KanMX cassette resistance marker flanked by *lox*P sequences to facilitate cloning using the Cre-*lox*P system was constructed by amplifying the cassette from p417-kan plasmid [32]. The cassette was amplified in two fragments that were overlapped by PCR and then cloned into pYTK001.

The hygromycin resistance marker in plasmid pUCC001 was changed to the nourseothricin resistance gene (Nat) using the In-Fusion HD Cloning Kit (Takara Bio, Kusatsu, Shiga Prefecture, Japan) at a ratio of 2:1 (insert:vector), resulting in plasmid pUCC001-v2. Cloning of a gRNA targeting the *URA3* gene into pUCC001-v2 was performed according to [33].

Briefly, to construct pSphI-*PaISPS*, plasmid pSphI was initially linearized with *Hind*III-HF (New England Biolabs, Ipswich, MA, USA) and treated with Klenow fragment of DNA polymerase I (NZYTech, Lisbon, Portugal) to generate blunt ends. Blunted vector was then treated with FastAP Thermosensitive Alkaline Phosphatase (Thermo Scientific, Massachusetts, USA). PCR-amplified gene expression cassette from pUDE-*PaISPS* was treated with *Dpn*I (Thermo Scientific, Waltham, MA, USA) and then phosphorylated with T4 polynucleotide kinase (Thermo Scientific, Waltham, MA, USA). Blunt-end ligation was performed overnight at 15 °C using a ratio of 1:5 and 50 ng of vector.

Strain YBL001-004 was constructed by integrating two copies of the *PaISPS* gene in tandem with mVenus as a reporter gene into the *lac4* locus (MB-pUDI-008 cassette) and intergenic region I2 (MB-pUDI-009) [27]. Strain YBL0001-006 was built by sequential integration of MB-pUDI-008 cassette, another copy of the *PaISPS* gene in tandem with native *IDI1* gene (MB-pUDI-010) into the intergenic region I2, and a copy of native *ERG10* gene in tandem with *S. cerevisiae HMG2* mutant (K6R mutation) (MB-pUDI-011) into the intergenic region I4. The KanMX marker was removed using the Cre-lox system [34]. Strain YBL001-007 resulted from *URA3* gene deletion in YBL001-006 by CRISPR-Cas9 system using plasmid pUCC001-v2-gURA3 and a cassette composed of 513 bp upstream and 500 bp downstream homology arms overlapped by PCR [33]. Strain YBL001-011 was built by transforming YBL00-007 with plasmid pSphI-*PaISPS*.

### 2.3. Eucalyptus globulus Wood (EGW) Cellulosic Fraction Saccharification

A solid fraction (cellulosic) previously obtained from *E. globulus* wood hydrothermal pretreatment was used in this work. The composition of the hydrothermally pretreated EGW, previously determined by [35], was (measured as g/100 g of pretreated EGW in oven dry basis ± standard deviation): 59 ± 0.23% of glucan, 2.1 ± 0.11% of xylan, and 34 ± 0.40% of Klason lignin. Saccharification was performed on 10% solids loading in 0.05 M of sodium citrate buffer pH 4.8, with the addition of cellulase (Cellic CTec2) at 20 FPU of cellulase/g of pretreated EGW to a final volume of 100 mL for 72 h, 150 rpm, at 50 °C. Cellic Ctec2 was kindly provided by Novozyme (Bagsvaerd, Denmark), and its activity (122 FPU mL^−1^) was measured by the method described by [36].

### 2.4. Fermentation Assays

Analysis of isoprene production by strains YBL001-004 and YBL001-006 was assessed at 30 °C, 200 rpm, and an initial OD_600nm_ of 0.1 for 48 h in 2 mL of YPD in 20 mL sealed serum bottles with the addition of 1.5 bar of pure oxygen in the beginning of the fermentation. Isoprene was produced in sealed serum bottles since its imprisonment is facilitated, thereby avoiding losses by evaporation. Optimization of isoprene specific titres in YPD was carried out at 30 °C, 200 rpm, and an initial OD_600nm_ of 0.1 for 48 h at different headspace-to-culture ratios for strain YBL001-006 (Supplementary Table S4). All fermentations were performed with two biological duplicates.

Fermentations under microaerobic to anaerobic conditions of EGW cellulosic hydrolysate (9.51 mL) by strain YBL001-011 were performed under condition C2.3 (39:1 ratio in 580 mL serum bottles; Supplementary Table S4) at 30 °C and 200 rpm, supplemented with 2.32 mL of SD medium (without carbon source) using an inoculum of 8 g L^−1^ of cells. Microaerobic to anaerobic conditions represent the transition from low oxygen concentration at atmospheric pressure and 30 °C (ca. 7.54 mg L^−1^) on the sealed flask to the exhaustion of oxygen. To assess plasmid loss percentage after 48 h, cells were plated in YPD at a dilution of 1:1000. One day later, 100 colonies were picked (in duplicate) onto SD medium plates. Headspace samples of 500 µL were taken for GC-MS analysis of isoprene production, and liquid culture samples were retrieved for HPLC analysis.

### 2.5. Analytical Methods

Fermentation samples were analyzed for quantification of metabolites by HPLC using an Aminex HPX-87H column (Bio-Rad, Hercules, CA, USA), operating at 60 °C, with a mobile phase of 5 mM H_2_SO_4_ (Fisher Scientific, Waltham, MA, USA) and flow rate of 0.6 mL min^−1^. Peaks were detected using a refractive index detector. For isoprene analysis in the headspace, sealed bottles were heated for 40 min at 40 °C at 200 rpm, and 500 µL of headspace was collected using a gas-tight syringe and analyzed by GC-MS. The system consisted of a SCION 436-GC-MS system (Scion Instruments, Fulton, MD, USA) equipped with a Rxi-5Sil MScolumn (30 m × 0.25 mm; 0.25 µm film thickness; Restek, Bellefonte, PA, USA) and using helium as the carrier gas. The oven temperature program was as follows: the initial temperature was 40 °C and then increased from 40 to 45 °C at 5 °C min^−1^, from 45 to 50 °C at 2 °C min^−1^, and finally from 50 °C to 200 °C at 20 °C min^−1^. The injector was maintained at 150 °C. The peak area was converted to isoprene mass (mg) using a calibration curve. Calibration curves were prepared by diluting different gas volumes (50, 100, 150, 200, and 250 µL) to a final volume of 500 µL, using a gas-tight syringe (Hamilton Company, Nevada, USA), from a heated 0.001 or 0.01 g L^−1^ solution of pure isoprene (Sigma-Aldrich, St. Louis, MO, USA) prepared in water (50 mL in 250 mL sealed bottles). Biomass dry cell weight (g L^−1^; Equation (1)) was quantified by collecting at least 1 mL of broth in previously dried and weighted tubes. The pellet was washed with deionized water, incubated at 105 °C for 24 h, and weighed again.

(1) $$\frac{Weigh\;of\;dried\;tube\;with\;biomass\left(g\right)-Weigh\;of\;empty\;dried\;tube\left(g\right)}{Volume\;of\;broth\;(L)}$$

### 2.6. Statistical Analysis

Statistical analysis was conducted using two-way ANOVA and the Tukey post hoc tests in GraphPad Prism for Windows version 8.0.2. Statistical significance was considered at a *p* value < 0.05, with “ns” denoting nonsignificant results (*p* value > 0.05), * indicating *p* value < 0.05, ** denoting *p* value < 0.01, *** indicating *p* value < 0.001, and **** denoting *p* value < 0.0001.

## 3. Results and Discussion

### 3.1. Metabolic Engineering Strategies for Improved Isoprene Biosynthesis from Glucose in K. marxianus

**Figure 1:**
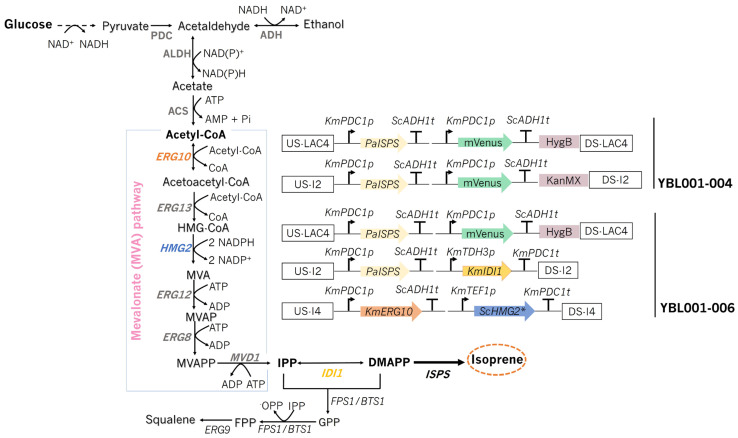
MVA pathway for generation of isoprene precursors and metabolic engineering strategies for improved isoprene production. Dotted arrows not all the metabolic steps are shown. Schemes of genetic modifications performed to generate strains YBL001-004 and YBL001-006 are represented. Genes of enzymes: *ERG10*, acetoacetyl-CoA thiolase; *ERG13*, 3-hydroxy-3-methylglutaryl-CoA (HMG-CoA) synthase; *HMG2*, HMG-CoA reductase; *HMG*2*, mutated variant; *ERG12*, mevalonate kinase; *ERG8*, phosphomevalonate kinase; *MVD1*, mevalonate pyrophosphate decarboxylase; *IDI1*, IPP isomerase; *ISPS*, isoprene synthase; *FPS1*, farnesyl pyrophosphate synthetase; *BTS1*, geranylgeranyl diphosphate synthase. Metabolites abbreviations: IPP, isopentenyl diphosphate; DMAPP, dimethylallyl diphosphate; HMG-CoA, 3-hydroxy-3-methylglutaryl-CoA; MVA, mevalonate; MVAP, mevalonate 5-phosphate; MVAPP, mevalonate 5-diphosphate, GPP, geranyl pyrophosphate; FPP, farnesyl diphosphate. Abbreviations of enzymes: PDC, pyruvate decarboxylase; ADH, alcohol dehydrogenase; ALDH, aldehyde dehydrogenase; ACS, acetyl-CoA synthetase.

Isoprene production in yeast can be enabled heterologous expression of a plant-derived gene encoding isoprene synthase (IspS). The IspS from *Populus alba* (PaISPS) (Uniprot Accession: Q50L36) was chosen because it has previously been successfully expressed in both yeast and bacteria. By expressing one copy of the *ISPS* gene, isoprene was undetected. A high gene copy number can result in higher enzymatic activity, which could, in this case, facilitate isoprene detection [37]. Moreover, high *ISPS* gene copy number could help reduce DMAPP intermediate toxicity by pushing the metabolism towards isoprene production [38,39]. As such, two copies of the *PaISPS* gene were cloned in tandem with mVenus, both under the control of the native strong promoter *KmPDC1*p and *S. cerevisiae* terminator *ScADH1*t, resulting in strain YBL001-004 (Figure 1).

**Figure 2:**
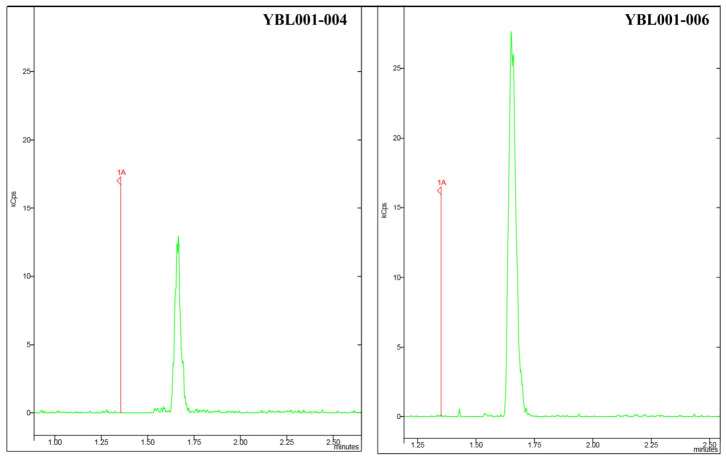
Isoprene production by strains YBL001-004 and YBL001-006. Fermentations were performed at 30 °C, 200 rpm, and an initial OD_600nm_ of 0.1 for 48 h in 2 mL of YPD in 20 mL sealed serum bottles with the addition of 1.5 bar of pure oxygen in the beginning of the fermentation. Green peaks correspond to the isoprene peak filtered for the predominant ion (67).

Isoprene production by strain YBL001-004 was detected in the gas phase from fermentations under microaerobic conditions in YPD medium in the presence of extra oxygen, but its quantification was not possible, probably due to metabolic limitations (Figure 2).

The supply of IPP and DMAPP, isoprene and sterols precursors, via the MVA pathway is tightly regulated at both transcriptional and post-transcriptional levels. In *S. cerevisiae*, the rate-limiting enzyme of the pathway, HMGR, comprises two isozymes (HMG1p and HMG2p) that are differentially feedback-regulated [40]. In *K. marxianus*, only the HMG1p coding gene was found. Moreover, pulling acetyl-CoA to the MVA pathway can contribute to increased isoprene production [41]. To understand if isoprene production was being affected by limited precursors supply, we overexpressed selected genes of the MVA pathway together with two copies of the *PaISPS* gene, thereby generating strain YBL001-006 (Figure 1). Those genes included *IDI1*, which catalyzes the isomerization between IPP and DMAPP and can therefore influence the balance of these intermediates, potentially increasing DMAPP accumulation; *ERG10*, whose overexpression may enhance the pull of acetyl-CoA into the mevalonate (MVA) pathway; and a copy of a mutated version of the *S. cerevisiae HMG2* gene, encoding the rate-limiting step of the MVA pathway (Figure 1). This mutated HMG2 variant is known not to interfere with yeast growth and was expected to stabilize the enzyme, thereby reducing its degradation [42].

Isoprene production by YBL001-006 was detected, and the peak area was approximately 2.5-fold higher than for strain YBL001-004 under the same fermentation conditions (Figure 2), indicating the positive effect of pushing isoprene precursors. The low dry cell weight (DCW) attained by strains YBL001-004 (3.45 ± 0.45 g L^−1^) and YBL001-006 (5.30 ± 0.40 g L^−1^) is likely to affect isoprene production as a result of lower levels of ATP and NADPH, required in the MVA pathway. Considering that *K. marxianus* is generally considered a Crabtree-negative yeast that benefits from high oxygen availability for optimal biomass formation and respiratory metabolism, oxygen limitation in small, sealed flasks may contribute to reduced growth and, consequently, low isoprene titres.

Further fine-tuning of metabolic engineering strategies to improve titres could build on the trends observed in this study. In particular, increasing the gene copy number of the isoprene synthase represents a promising approach. In addition, modifications to the MVA pathway to enhance metabolic flux may be achieved through overexpression of genes encoding rate-limiting steps (e.g., a mutant *ScHMG2* or truncated *KmHMG1*) [43]. Improving cytosolic acetyl-CoA supply is another potential target, either through the expression of heterologous pathways [44,45], reduction of competing pathways such as acetyl-CoA transport to the peroxisome [46], or downregulation of genes involved in sterol biosynthesis [41,47]. Moreover, overexpression of key genes involved in fatty acid β-oxidation has been shown in *S. cerevisiae* to enhance the cytosolic acetyl-CoA pool and increase production of compounds such as (2S)-naringenin [48]. More recently, channelling peroxisomal acetyl-CoA via mitochondrial/peroxisomal carnitine acetyl-CoA transferase (Cat2) in *K. marxianus* has been reported to improve cytosolic acetyl-CoA availability and increase triacetic acid lactone production [49].

### 3.2. Optimization of Fermentation Conditions Enhances Isoprene Production in K. marxianus

**Figure 3:**
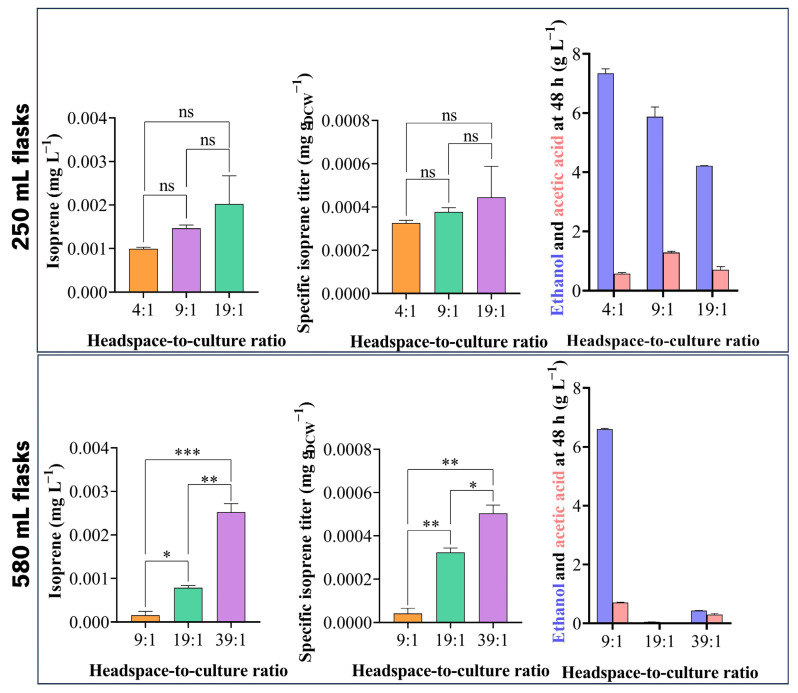
Headspace-to-culture ratios tested for improved isoprene production in sealed serum bottles. Headspace-to-culture ratios of 4:1, 9:1, and 19:1 were tested in 250 mL serum bottles, while ratios of 9:1, 19:1, and 39:1 were tested in 580 mL serum bottles. Fermentations were carried out at 30 °C and 200 rpm, in YPD medium for 48 h with strain YBL001-006. Each bar represents the average and error bars of standard deviation of biological duplicates. Statistical significance was considered at a *p* value < 0.05, with “ns” denoting nonsignificant results (*p* value > 0.05), * indicating *p* value < 0.05, ** denoting *p* value < 0.01, and *** indicating *p* value < 0.001.

As isoprene quantification was hindered at low culture volumes, likely due to limited oxygen supply constraining cell growth, different headspace-to-culture ratios were tested to address this limitation (Figure 3). The headspace volume has proven to significantly impact the production of volatile organic compounds under anaerobic conditions [50]. Headspace and culture volumes should be carefully balanced to ensure sufficient oxygen availability in the headspace for cell growth and, at the same time, allow efficient isoprene detection after vaporization. Furthermore, balancing headspace-to-culture volume may be more cost-effective than introducing oxygen in the culture to further improve isoprene production.

No significant differences (*p* value > 0.05) were observed in isoprene absolute and specific titre for strain YBL001-006 between the three headspace-to-culture volume ratios tested in 250 mL serum bottles, although an increasing trend with higher ratios was noted.

In contrast, fermentations in 580 mL serum bottles enabled more robust discrimination between conditions and the evaluation of higher headspace-to-culture ratios. A 7.7-fold significantly higher (*p* value < 0.01) specific isoprene titre was observed for ratio 19:1 compared to 9:1, a 1.6-fold significantly higher (*p* value < 0.05) specific isoprene titre was observed for ratio 39:1 compared to 19:1, and a 12-fold significantly higher specific isoprene titre was observed for ratio 39:1 compared to 9:1 (*p* value < 0.01). Ratio 39:1 resulted in higher specific isoprene titres, indicating that higher headspace volume favoured isoprene production and detection (Figure 3). In 250 and 580 mL serum bottles, higher headspace-to-culture ratios led to increased biomass (DCW; Table 1) and specific isoprene titres, indicating that improved oxygen availability supported growth and production.

When using serum bottles of 250 and 580 mL of total volume, the condition with higher headspace-to-culture ratio resulted in lower accumulation of ethanol at 48 h (Figure 3). The condition of 9:1 headspace-to-culture ratio resulted in higher accumulation of acetic acid at 48 h, regardless of the total flask volume (Figure 3). As such, these conditions would probably not favour isoprene production, since acetic acid was not converted to acetyl-CoA. Overall, a ratio of 39:1 in 580 mL serum bottles was used for further experiments, since it resulted in low accumulation of metabolites after 48 h and higher specific isoprene titres.

At industrial scale, gas–liquid mass transfer, reactor design, and off-gas handling become dominant factors in volatile product recovery. The observed relationship between headspace volume and isoprene accumulation in sealed bottles may not translate directly to larger bioreactors where continuous gas stripping, pressure control, and condensation systems are typically used to capture isoprene. However, the flask-level results still offer useful guidance for understanding the partitioning behaviour of isoprene, which can inform process engineering decisions such as gas flow rates and reactor venting design.

### 3.3. Isoprene Biosynthesis from the Cellulosic Fraction of Pretreated Eucalyptus globulus by Engineered K. marxianus

Considering that increasing the number of copies of the *PaISPS* gene may contribute to higher isoprene production together with pulling the metabolic flux through the MVA pathway, we transformed strain YBL001-007 (derived from YBL001-006 with *URA3* gene deletion) with a multicopy plasmid containing pKD1 replication origin expressing *PaISPS* gene under the control of the native strong promoter *KmPDC1*p and *S. cerevisiae* terminator *ScADH1*t, thereby generating strain YBL001-011.

**Figure 4:**
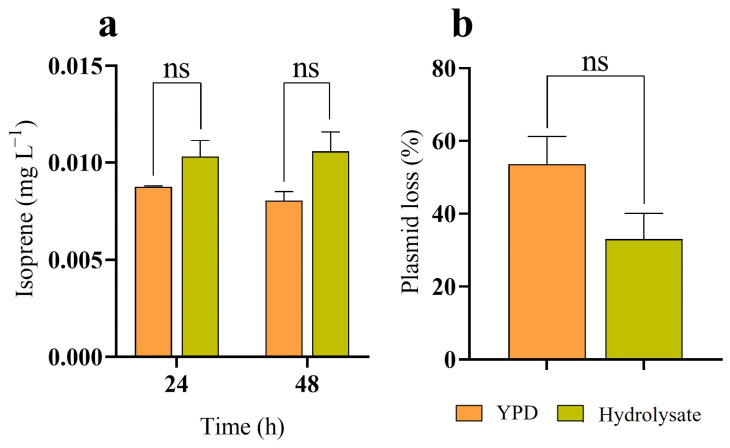
Isoprene production by strain YBL001-011. (**a**) Isoprene titres after 24 and 48 h and (**b**) percentage of plasmid loss after 48 h from the cellulosic fraction of autohydrolyzed EGW (green bars) and YPD (orange bars) at 30 °C and 200 rpm under condition C2.3 (39:1 ratio in 580 mL serum bottles; Supplementary Table S4). Each bar represents the average and error bars of standard deviation of biological duplicates. Statistical significance was considered at a *p* value < 0.05, with “ns” denoting nonsignificant results (*p* value > 0.05).

Microbial production of isoprene from lignocellulosic feedstocks remains largely unexplored, to the best our knowledge, and it could contribute to the development of an environmentally sustainable process. Considering that glucose can be obtained from the cellulosic fraction of lignocellulosic biomass after pretreatment, autohydrolyzed *E. globulus* wood (EGW) was used for isoprene production given its high cellulose content. Developing a process for EGW valorization involves separate hydrolysis of cellulose in the pretreated solid under aerobic conditions, which favour cellulases activity, to release glucose prior to fermentation under anaerobic conditions. After 72 h of saccharification, 15.52 g L^−1^ of glucose and 2.96 g L^−1^ of xylose were released from the solid. The cellulosic hydrolysate was used for isoprene production by strain YBL001-011, supplemented with SD medium without carbon source. Fermentations were also conducted using YPD for comparison (Figure 4).

Isoprene production from YPD by strain YBL001-011 was not significantly different (*p* value > 0.05) from that obtained using the cellulosic hydrolysate, although the final titre from YPD was 1.3-fold lower. Maximum isoprene titre produced from YPD was attained after 24 h (9 μg L^−1^), while from the cellulosic hydrolysate, the maximum was achieved at 48 h (11 μg L^−1^) (Figure 4a). It is worth noting that some plasmid loss was observed in strain YBL001-011, suggesting that higher titres could potentially be achieved. No statistically significant differences (*p* value > 0.05) in plasmid loss were detected after 48 h of fermentation between YPD and the cellulosic hydrolysate (Figure 4b).

Fermentation in YPD resulted in the accumulation of 1.49 ± 0.08 g L^−1^ of ethanol and 1.21 ± 0.01 g L^−1^ of acetic acid. In contrast, no accumulation of these metabolites was observed during fermentation of the cellulosic hydrolysate, suggesting that their conversion to acetyl-CoA may have contributed to isoprene production. No accumulation of xylitol was observed during fermentation of the cellulosic hydrolysate, and the carbon sources, glucose and xylose, were no longer detected after 24 h.

## 4. Conclusions

In this study, isoprene production from glucose by *K. marxianus* in sealed flasks supplemented with extra oxygen was demonstrated for the first time using a strain expressing two copies of an isoprene synthase gene from *P. alba.* Metabolic engineering strategies aimed at enhancing precursors flux through the MVA pathway were implemented, resulting in a 2.5-fold increase in isoprene peak area under the same fermentation conditions. However, absolute quantification was not possible at that small scale (20 mL). Our results also highlighted the importance of optimizing the headspace-to-culture ratio for small-scale isoprene production. The best-performing condition was achieved at a 39:1 ratio in 580 mL flasks, yielding 2.53 μg L^−1^ of isoprene. Furthermore, isoprene production was improved using a strain carrying a multicopy plasmid and cultivated on a lignocellulosic feedstock, despite the more stressful conditions for yeast, reaching titres of 11 μg L^−1^.

Overall, these results suggest that increasing gene copy number contributes to enhanced isoprene production, although direct quantification of isoprene synthase expression was not performed and should be addressed in future studies to better establish this relationship. This work therefore represents an initial step toward the development of sustainable biorefineries based on *K. marxianus*. From a process perspective, implementing fed-batch fermentations in controlled bioreactors with enhanced oxygen transfer and efficient gas stripping would likely improve both isoprene production and recovery. From a metabolic engineering standpoint, in addition to optimizing gene copy number, strategies such as enhancing precursor supply (e.g., increasing flux through the MVA pathway), fine-tuning isoprene synthase expression, exploring enzymes from different sources, and improving cofactor balance represent promising avenues for further improvement.

## Figures and Tables

**Table 1:** Dry cell weight obtained at different headspace-to-culture ratios evaluated for improved isoprene production in sealed serum bottles. Ratios of 4:1, 9:1, and 19:1 were tested in 250 mL serum bottles, while ratios of 9:1, 19:1, and 39:1 were evaluated in 580 mL bottles. Fermentations were performed with strain YBL001-006 in YPD medium at 30 °C and 200 rpm for 48 h. Data are presented as mean ± standard deviation of biological duplicates.

Flask Volume (mL)	Headspace-to-Culture Ratio	Dry Cell Weight (g L^−1^)
250	4:1	3.05 ± 0.10
	9:1	3.89 ± 0.03
	19:1	4.54 ± 0.06
580	9:1	3.74 ± 0.05
	19:1	4.51 ± 0.04
	39:1	5.02 ± 0.93

## Data Availability

The original contributions presented in this study are included in the article/Supplementary Material. Further inquiries can be directed to the corresponding author.

## Supplementary Materials

The following supporting information can be downloaded at: https://www.mdpi.com/article/10.3390/jof12050343/s1, Table S1. *K. marxianus* strains used in this study; Table S2. List of plasmids used in this work. *Not*I recognition sites used for the digestion of the integrative cassettes are indicated in superscript. *Bsa*I restriction sites for cloning of a gRNA are also indicated in superscript. Abbreviations: *Saccharomyces cerevisiae* (Sc); *Kluyveromyces marxianus* (Km); US/DS, upstream and downstream homology arms, respectively. Table S3. Oligonucleotides used in this work. *Bsm*BI (blue) and *Bsa*I (green) recognition sites are highlighted. Abbreviations: *Saccharomyces cerevisiae* (Sc); *Kluyveromyces marxianus* (Km). Table S4. Headspace-to-culture ratios tested for isoprene production in sealed flasks.

## Abbreviations

The following abbreviations are used in this manuscript:

DCW	Dry cell weight
DMAPP	Dimethylallyl diphosphate
FPP	Farnesyl diphosphate
GPP	Geranyl diphosphate
IPP	Isopentenyl diphosphate
IspS	Isoprene synthases
MVA	Mevalonate
YNB	Yeast nitrogen base without amino acids
YPD	Yeast extract–peptone–dextrose
