# Supplementary material for: Metabolic Engineering Kluyveromyces marxianus for Isoprene Production from Eucalyptus globulus Wood Cellulosic Fraction
Source: J Fungi (Basel). 2026 May 6;12(5):343. doi: 10.3390/jof12050343 (PMC13208150; doi:10.3390/jof12050343)
Supplement: Supplementary file 1 [file jof-12-00343-s001.zip › jof-4267415-supplementary.pdf]

Supplementary material

Metabolic engineering *Kluyveromyces marxianus* for isoprene production from *Eucalyptus globulus* wood cellulosic fraction

Table S1. *K. marxianus* strains used in this study.

Strain	Genotype	Resistance/Auxotrophy	Ref
YBL001	NBRC1777 *yku80-1*		Rajkumar et al., 2019
YBL001-004	YBL001-*lac4:: PaISPS*- mVenus, I2:: *PaISPS*-mVenus	Hygromycin B, KanMX	This study
YBL001-006	YBL001-*lac4::PaISPS*- mVenus, I2:: *PaISPS*-KmIDI1, I4::*KmERG10*-*ScHMG2*	Hygromycin B	
YBL001-007	YBL006 Δ*ura3*	Uracil	
YBL001-011	YBL001-007 harbouring plasmid pSphI-*PaISPS*		

Table S2. List of plasmids used in this work. *Not*I recognition sites used for the digestion of the integrative cassettes are indicated in superscript. *Bsa*I restriction sites for cloning of a gRNA are also indicated in superscript. Abbreviations: *Saccharomyces cerevisiae* (*Sc*); *Kluyveromyces marxianus* (*Km*); US/DS, upstream and downstream homology arms, respectively.

**Plasmid name**	**Description**	**Ref**
pGGKd015	Ori, ampR, ConLS, sfGFP, ConR1	Hassing et al., 2019
pGGKd072	Ori, ampR, ConL1, sfGFP, ConRE, *ScURA3*, 2µ ori	Hassing et al., 2021
pGGkd068	Ori, kanR, ^{NotI}Kmlac4-US, ConRE', sfGFP, ConLS, hygB, Kmlac4-DS^{NotI}	Dekker et al., 2021
pKlNatCre	Ori, ampR, KlCEN2, ARSHCEN6, KARS, *AgTEF1p-Nat-AgTEF1t, ScGAL1p-Cre-ScCYC1t*	Ribeiro et al., 2007
p417-kan	Ori, ampR, 2µ ori, *ScTEF1p-GRE3-ScTDH3t*, *lox*P-*AgTEF1p*-KanMX-*AgTEF1t*-*lox*P	Baptista et al., 2018
pSphI	Ori, ampR, pKD1, *ScURA3*	Panuwatsuk & Da Silva, 2002
pUDE-*PaISPS*	Ori, ampR, ConLS, *KmPDC1p*-*PaISPS*-*ScADH1t*, ConR1	This study
pUDE-mVenus	Ori, ampR, ConL1, *KmPDC1p*-mVenus-*ScADH1t*, ConRE, *ScURA3*, 2µ ori	
pUDE-*KmIDI1*	Ori, ampR, ConL1, *KmTDH3p*-*KmIDI1*-*ScADH1t*, ConRE, *ScURA3*, 2µ ori	
pUDE-*KmERG10*	Ori, ampR, ConLS, *KmPDC1p*-*KmERG10*-*ScADH1t*, ConR1	
pUDE-*ScHMG2*	Ori, ampR, ConL1, *KmTEF1p*-*ScHMG2*-*KmPDC1t*, ConRE, *ScURA3*, 2µ ori	
pUDI-I2	Ori, kanR, ^{NotI}I2-US, ConLS'-sfGFP-ConRE', KanMX, I2-DS^{NotI}	
pUDI-I2-v2	Ori, kanR, ^{NotI}I2-US, ConLS'-sfGFP-ConRE', *lox*P-KanMX-*lox*P, I2-DS^{NotI}	
pUDI-I4	Ori, kanR, ^{NotI}I4-US, ConLS'-sfGFP-ConRE', *lox*P-KanMX-*lox*P, I4-DS^{NotI}	
MB-pUDI-008	Ori, kanR, ^{NotI}Kmlac4-US, *KmPDC1p*-*PaISPS*-*ScADH1t*, *KmPDC1p*-mVenus-*ScADH1t*, hygB Kmlac4-DS^{NotI}	

MB-pUDI-009	Ori, kanR, ^{NotI}I2-US, *KmPDC1p-PaISPS-ScADH1t*, *KmPDC1p*-mVenus-*ScADH1t*, KanMX, I2-DS^{NotI}
MB-pUDI-010	Ori, kanR, ^{NotI}I2-US, *KmPDC1p-PaISPS-ScADH1t*, *KmTDH3p-KmIDI1-KmPDC1t*, *lox*P-KanMX-*lox*P, I2-DS^{NotI}
MB-pUDI-011	Ori, kanR, ^{NotI}I4-US, *KmPDC1p-KmERG10-ScADH1t*, *KmTEF1p-ScHMG2-KmPDC1t*, *lox*P-KanMX-*lox*P, I4-DS^{NotI}
pUCC001-v2	Ori, ampR, panARS(OPT), *AgTEF1p*-Nat-*AgTEF1t*, *ScTDH3p*$^{\mathrm{BsaI\ BsaI}}$*ScCYC1t*, *AaTEF1p-Spcas9*$^{D147Y\ P411T}$*-ScPHO5t*
pUCC001-v2-gURA3	Ori, ampR, panARS(OPT), *AgTEF1p-Nat-AgTEF1t*, *ScTDH3p*-gURA3-*ScCYC1t*, *AaTEF1p-Spcas9*$^{D147Y\ P411T}$*-ScPHO5t*
pSphI-*PaISPS*-v2	Ori, ampR, pKD1, *KmPDC1*p-*PaISPS*-v2-*ScADH1t*, *ScURA3*

Table S3. Oligonucleotides used in this work. *Bsm*BI (blue) and *Bsa*I (green) recognition sites are highlighted. Abbreviations: *Saccharomyces cerevisiae* (Sc); *Kluyveromyces marxianus* (Km).

Primer name	Sequence	Purpose
Palba-ISPS-FW	GCATCGTCTCATCGGTCTCATATGTGTAGTGTTTCTACTGAGAATG	Amplification of *Populus alba ISPS* gene (*PaISPS*)
P.alba-ISPS-RV	ATGCCGTCTCAGGTCTCAGGATCCTCATCTCTCAAAAGGAAGGATAG	
pYTK001-FW	TTTGCTGGCCTTTTGCTC	Colony PCR primers to confirm cloning into pYTK001
pYTK001-RV	CGTGGCGCTTTCTCATAG	
hygB-RV	ATTGTCCGTCAGGACATTG	Colony PCR reverse primer, priming on hygromycin B resistance marker
pYTK001_check_RV	CTGTGGATAACCGTAGTCGG	Sequencing primer for confirmation of cloning into pYTK001
ASR_K001-RV	ATTGGTAACTGTCAGACCAAGTTTA	Colony PCR reverse primer, priming on the end of ampR
ConR1-RV	AGTCATCCGAGCGTGTATTG	Colony PCR reverse primer, priming on ConR1
KanMX-check-RV	GGGCGACAGTCACATCATG	Sequencing primer, priming at the 3' end of KanMX
Bf-ConL1-RV	GCTGGCCTTTTGCTCACATG	Sequencing primer, priming before the 3' end of ConL1
URA-check-RV	TGCTCCTTCCTTCGTTCTTC	Colony PCR primer, priming on pGGKd072 plasmid 132 bp upstream of the *ScURA3*
ScADH1t-RV	CAATAAGAGCGACCTCATG	Colony PCR and sequencing primer, priming at the 3' end of the *S. cerevisiae ADH1* terminator
KmIDI1-FW	GCATCGTCTCATCGGTCTCATATGTCTACGGAGACGTCAACTTAC	Amplification of the *KmIDI1* gene (chr. 5)
KmIDI1-RV	ATGCCGTCTCAGGTCTCAGGATCCTCACAACATTCTATGGATTTCAG	
Km_I2-Right-FW	AAGCATCGTCTCATCGGTCTCAGAGTTGAAGCATAGAAGAAATGTTTAACCA	Amplification the right homology arm for I2 intergenic region (1002 bp)

Km_I2-Right-RV	TTATGCCGTCTCAGGTCTCATCGGGTTTTGGGGCAT TTAATAAGAATTTG	
Km_I2-Left-FW<br>Km_I2-Left-RV	AAGCATCGTCTCATCGGTCTCACAATACCGAATATT AAATAATCTTTTTTTATTTCTTAT<br>TTATGCCGTCTCAGGTCTCAAGGG ATCTCATCCTCTAATGACGTAAA	Amplification of left homology arm for I2 intergenic region (999 bp)
Km_I4-Right-FW<br>Km_I4-Right-RV	AAGCATCGTCTCATCGGTCTCAGAGTTTCACAATGG AGGGAGTG<br>TTATGCCGTCTCAGGTCTCATCGG TGTTGCCGGACACATATTAC	Amplification of right homology arm for I4 intergenic region (850 bp)
Km_I4-Left-FW<br>Km_I4-Left-RV	AAGCATCGTCTCATCGGTCTCACAATGGTGTTGCAT TAGTATCGC<br>TTATGCCGTCTCAGGTCTCAAGGGTGTTCATAAGAA CATGGTTTATGGA	Amplification of left homology arm for I4 intergenic region (850 bp)
KmERG10-FW<br>KmERG10-RV	GCATCGTCTCATCGGTCTCATATGAGTGACAACGTA TACATTG<br>ATGCCGTCTCAGGTCTCAGGATCCTTAAACTCTTTC AATAACAATAGAAG	Amplification of the *KmERG10* gene (chr. 6)
FW_amdS_check	TCTGGGCAGATGATGTCGAG	Colony PCR reverse primer, priming at the 5' end of the *Ashbya gossipii TEF1* terminator
ASR_I1_US_FW<br>ASR_I1_DS_RV	CATTAGAACCTTTTTCAACACTC<br>CTTAGTGGTTGTGAAGGTTT	Confirmation of integrations into the *lac4* locus
ASR_I2-L_FW<br>ASR_I2-R_RV	GATAATAAGCTACCTGCTGA<br>AGTCCATTTTCTACATTTG	Confirmation of integrations into the I2 intergenic region
ASR_I4-L_FW<br>ASR_I4-R_RV	GTTTGTTAGTTAATATAGTAATACTATAGTAC<br>CAGAAAAGGCATTTGTGT	Confirmation of integrations into the I4 intergenic region
KanMX-OL-FW<br>KanMX-OL-RV	GCGTATTTCGTCTAGCTCAGG<br>CCTGAGCTAGACGAAATACGC	Overlap extension PCR internal primers for correction of internal *Bsm*BI site present in the KanMX cassette (from p47-kan plasmid)

KanMX-pYTK001-FW<br><br>KanMX-pYTK001-RV	AAGCATCGTCTCATCGGTCTCATACAAGGTCGACA ACCCTTAAT<br>TTATGCCGTCTCAGGTCTCAACTCGATCTGATATCA CCTAATAACTTCG	Amplification of the KanMX cassette flanked by *lox*P sequences without internal *Bsm*BI site
CloNAT_IF_FW<br><br>CloNAT_IF_RV	GATCATCAATAGGCAAGCTTGCCTCGTCCCCGC<br><br>CCTCTCGAAAGGTGACAGTATAGCGACCAGCATTC ACATAC	Amplification of the Nat marker from pYTK078
pUCC001-FW<br><br>pUCC001-RV	TCACCTTTCGAGAGGACGATG<br><br>TGCCTATTGATGATCTGGCGGAATG	Amplification of pUCC001 backbone, except hygromycin B marker
CloNAT_confir	TACGAAAGTTCTTGAATCTGG	Colony PCR primer for confirmation of substitution of hygromycin B to Nat marker in pUCC001
ScHMG2-FW<br><br>ScHMG2-RV	GCATCGTCTCATCGGTCTCATATGTCACTTCCCTTA AGAAC<br>ATGCCGTCTCAGGTCTCAGGATCCTTATAATAATGC TGAGGTTTTACAG	Amplification of the *S. cerevisiae* CEN.PK 113-11C *HMG2* mutant gene (chr. 12) for cloning into pYTK001
HMG2-SD-FW<br><br>HMG2-SD-RV	[Pho]TCTATAGTAGTGTGTGCATTTTTC<br><br>TCTCCTGTGTCTGATACATG	Site directed mutagenesis primers for correction of *ScHMG2* internal *Bsa*I restriction site
gURA3-FW<br><br>gURA3-RV	CGTCAGGTTCTTTCGTAACTTCCT<br><br>AAACAGGAAGTTACGAAAGAACCT	Amplification of the guide RNA targeting the *KmURA3* gene
Bsa-RV	TACACGCGTTTGTACAGAAAAAAAAGAAAAATTTG A	Reverse primer to check for correct assembly of gRNA expression vector – pUCC001 - together with gURA3-FW primer
US-URA3-FW<br><br>US-URA3-RV	ATCAAAACTGAAATTAGGTGCCTG<br><br>TCTTAGTCGACATCCTCCTTTGATTAG	Amplification of a 526 bp homology arm upstream of the *KmURA3* gene

DS-URA3-FW	ATAAACTAATCAAAGGAGGATGTCGACTAAGAGTTCTCCGAGAACAAG	Amplification of a 530 bp homology arm downstream of the *KmURA3* gene
DS-URA3-RV	TATAGACGCATGAAGTCCTTC	
ISPS-GEC-FW	TCAACGCATATAGCGCTAATCCAGCGAATATACAGCGTG	Amplification of gene expression cassette from pUDE-*PaISPS*
ISPS-GEC-RV	GTCACTATGGCGTGCTGCGAAATGGGGAGCGATTTG	

Table S4. Headspace-to-culture ratios tested for isoprene production in sealed flasks.

Condition	Headspce-to-culture ratio	Flask total volume (mL)	Culture volume (mL)
C1.1	4:1	250	50
C1.2	9:1		25
C1.3	19:1		12.5
C2.1	9:1	580	58
C2.2	19:1		29
C2.3	39:1		14.5
